# Perioperative serum cortisol levels in ACTH sufficient and ACTH deficient patients during transsphenoidal surgery of pituitary adenoma

**DOI:** 10.1007/s12020-018-1655-8

**Published:** 2018-07-02

**Authors:** Henrik Borg, Peter Siesjö, Babar Kahlon, Sigridur Fjalldal, Eva Marie Erfurth

**Affiliations:** 1 Department of Endocrinology, Skåne University Hospital, Lund, S-221 85 Sweden; 2 Department of Neurosurgery, Skåne University Hospital, Lund, S-221 85 Sweden

**Keywords:** Hydrocortisone, endoscopic transsphenoidal surgery, adrenal insufficiency; pituitary gland, adrenocorticotropic hormone, remifentanil

## Abstract

**Purpose:**

No previous study has analyzed serum cortisol levels during transsphenoidal endoscopic pituitary surgery in patients with and without hydrocortisone (HC) substitution.

**Methods:**

A total of 15 patients undergoing surgery for a pituitary adenoma were studied. Those with normal ACTH function were either not given HC (*n* = 7) or received 50 mg intravenous HC at the start of surgery (*n* = 4). Patients with ACTH deficiency received intravenous HC of 100 mg in the morning before surgery (*n* = 4) with the additional 50 mg for an afternoon operation (*n* = 2). Propofol and remifentanil were used as anesthetics. Serum cortisol was measured at the start of and every 30 min during surgery.

**Results:**

Among 7 patients with normal ACTH function without HC substitution, cortisol levels before surgery were 126–244 nmol/L, among the 4 patients undergoing surgery in the morning, whereas the 3 who underwent surgery in the afternoon had lower levels, 38–76 nmol/L. During nose/sinus surgery cortisol levels decreased to 79–139 and 24–54 nmol/L, respectively. At intrasellar manipulation a distinct rise was noted. Also, in the 4 ACTH sufficient patients receiving HC, cortisol levels decreased during nose/sinus surgery, but only with a slight increase during intrasellar surgery. In the 4 ACTH deficient patients cortisol peaked at 1914–2582 nmol/L.

**Conclusions:**

Patients with normal ACTH function without HC substitution had very low cortisol levels during the first part of surgery, likely suppressed by the anesthetics. After mechanical impact in the sella, a marked increase in cortisol was noted. Supraphysiological cortisol levels were achieved with our routine HC substitution, advising us to reduce the supplementation.

## Introduction

In 1952, a patient developed surgery-associated adrenal insufficiency as a result of preoperative withdrawal from glucocorticoid therapy [1]. That case report, and one other in the ensuing 12 months [2], prompted the publication of recommendations for perioperative glucocorticoid coverage, which became the standard care. Recommendation of glucocorticoid administration was then tapered depending on the magnitude of stress, from minor surgical stress (e.g., inguinal herniorrhaphy) to major surgical stress (e.g., pancreato-duodenectomy or cardiac surgery). Thus, higher glucocorticoid doses were provided for major surgical stress, e.g., 100–150 mg cortisone equivalent per day for 2–3 days.

The hormonal responses to surgery, critical illness, and trauma have been studied intensively. There is however a considerable inter-individual variation in the hypothalamic–pituitary–adrenal cortical (HPA) response to surgical stress [3]. Some of the variation can be attributed to the actions of premedication with benzodiazepines and of anesthetics [4], as the anesthesia during endoscopic pituitary surgery is often a combination of propofol and remifentanil. The latter is an ultrashort-acting opioid which reduces cortisol [5].

In recent years, patients with normal preoperative cortisol levels generally do not receive hydrocortisone (HC) during surgery for pituitary adenoma [6–8]. In some patients with suspected insufficiency there might be a borderline choice of whether HC should be provided or not. However, in some centers patients with normal serum cortisol are receiving HC, possibly just in case. All patients with normal adrenocorticotropic hormone (ACTH) function who undergo pituitary surgery at Skåne University Hospital are receiving peri- and immediate postoperative substitution with HC of 50 + 50 + 50 mg on the day of surgery. Among ACTH deficient patients the doses are 100 + 50 + 50 mg of HC.

Although the main reason to substitute during surgery is to avoid a possible postoperative deficiency, it may also protect against a perioperative decrease in cortisol levels. However, the serum cortisol levels in patients during pituitary surgery have previously not been analyzed.

Therefore, our aim was to measure the serum cortisol levels during endoscopic adenoma surgery in ACTH sufficient patients and among ACTH deficient patients during our routine HC substitution. We anticipated that ACTH sufficient patients have no need for any additional HC as they would remain ACTH sufficient during the operative procedure. Further, we suspected that our routine HC substitution would result in unnecessary high serum cortisol levels among ACTH deficient patients and therefore would need a reevaluation.

## Materials and methods

### Subjects

In total, 15 patients with surgery for a pituitary adenoma were studied. They were 28–84 years of age, and 12 were male. Seven had growth hormone (GH)-secreting adenomas, 1 had a mixed GH- and prolactin (PRL)-secreting adenoma, 1 had a prolactinoma, and 6 had non-functioning adenomas. The size of the adenomas ranged from 5 to 28 mm (Table 1).

**Table 1:** Perioperative hemodynamic parameters, vasopressor requirement, doses of anesthetics, and premedication in the ACTH sufficient patients

HC substit	Sex	Age (years)	Adenoma function	AD size (mm)	Start of surgery	Basal s-cortisol	BP (range)	HR (range)	Vasopressors	Propofol (mL)	Remifentanil (mL)	Premed midazolam (mg)
No	M	64	GH	7	09h20	238	130/80–70/50	70–85	No	112	55	No
No	F	39	PRL	7	13h15	76	110/70–70/45	55–70	No	85	71	No
No	M	51	GH	6	16h00	75	160/110–80/50	70–80	No	102	75	5
No	M	63	NF	15	09h45	126	175/90–90/45	50–75	No	116	56	No
No	M	27	GH	15	14h40	38	135/80–90/55	70–85	No	150	128	7.5
No	M	73	GH	5	09h25	244	130/60–90/45	50–55	No	77	80	5
No	M	53	NF	15	09h10	243	140/85–80/50	55–70	No	80	39	No
Yes	M	49	GH	11	09h20	407	110/60–85/50	60–65	No	86	88	No
Yes	M	33	GH	20	09h35	776	135/70–70/45	80–125	No	69	64	No
Yes	M	72	NF	24	09h10	1282	110/55–85/50	50–55	No	78	67	No
Yes	F	51	GH+PRL	17	09h40	1252	145/50–85/50	70–85	No	81	72	7.5

*HC substit* hydrocortisone substitution, *Adenoma function* NF non-functioning adenoma, *SC* serum cortisol, *BP* blood pressure, *HR* heart rate

The definition of ACTH sufficiency was based on cortisol testing some weeks before surgery and 11 patients had normal ACTH function, defined either as a serum cortisol in the morning >400 or >550 nmol/L after a 250 μg ACTH stimulation test. The ACTH sufficient patients, considered low risk for surgical complications, were sequentially assigned in a randomized manner to either the group with or without perioperative HC supplementation. Of the 11 patients, 7 were not given any HC supplementation, whereas 4 received the routine 50 mg intravenous HC shortly before the start of anesthesia and then 50 mg + 50 mg of HC during the following 12 h.

Four patients with confirmed ACTH deficiency received the routine intravenous HC; 2 received 100 mg in the morning before surgery, at 6–7 AM, and 2 received 100 mg in the morning plus 50 mg during surgery 8 h after the morning dose since they had the operation in the afternoon. Other hormone deficiencies were sufficiently substituted before surgery.

**Fig. 1:**
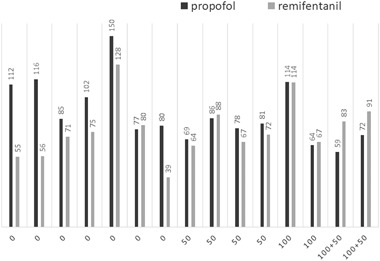
Doses of propofol (mL) and remifentanil (mL) in the patients without HC (*n* = 7), with 50 mg (*n* = 4), 100 mg (*n* = 2), and 100 + 50 mg (*n* = 2) HC, respectively

Serum cortisol was measured in blood samples collected just after the induction of anesthesia before the start of surgery (0 min) and every 30 min until the end of surgery. In transsphenoidal surgery at our institution, oto-laryngeal specialists operate in the nose and sinuses, and neurosurgeons operate in the sella. Surgery started between 9 AM and 4 PM, with 10 patients having surgery in the morning and 5 in the afternoon, and surgery lasted from 60 to 200 min. During hospitalization on the first day postoperatively (+1) and every morning thereafter before drug intake, serum cortisol levels were measured (data not shown). Premedication with midazolam was given to some of the patients (Table 1). The anesthetic method used was continuous infusion of propofol and remifentanil (Fig. 1, Table 1).

### Hormone assay

Serum cortisol was measured with an electrochemiluminescence immunoassay on a cobas analyzer (Roche Diagnostics, Mannheim, Germany). The normal reference range is 133–537 nmol/L for 06–10 AM, and is 68–327 nmol/L for 04–08 PM.

## Results

### ACTH sufficient patients (*n* = 7) not receiving HC substitution

**Fig. 2:**
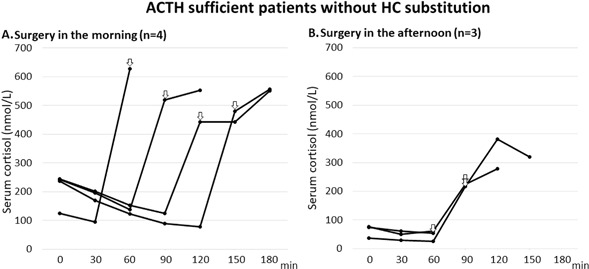
Serum cortisol levels in ACTH sufficient patients who did not receive any hydrocortisone substitution during pituitary surgery performed in the **a** morning (*n* = 4) and **b** afternoon (*n* = 3). Serum cortisol was measured from the start to the end of surgery. White arrows (⇩) indicate the first sample obtained after intrasellar manipulation (60–150 min)

The 4 patients undergoing surgery in the morning had basal serum cortisol levels starting at 126–244 nmol/L, whereas those undergoing surgery in the afternoon (*n* = 3) all had lower levels, 38–76 nmol/L (Fig. 2a, b). In the first part of the surgical procedure, during entrance to the nasal cavity and paranasal sinuses, cortisol levels decreased further to 79–139 and 24–54 nmol/L in the morning and afternoon groups, respectively (Fig. 2a, b). After surgery had reached into the sella turcica, rising cortisol levels were noted in 6 of 7 patients; rising with (Δ) 217–532 nmol/L up to a level of 278–628 nmol/L in cortisol. In those who had surgery in the morning (*n* = 4), cortisol levels rose with (Δ) 402–532 nmol/L (Fig. 2a). In 2 of the 3 patients who had surgery in the afternoon, cortisol rose with (Δ) 217 and 356 nmol/L, respectively, whereas in one patient in whom the duration of the operation was only 60 min no increase was recorded (Fig. 2b).

### Patients receiving HC substitution

#### ACTH sufficient patients receiving 50 mg HC

**Fig. 3:**
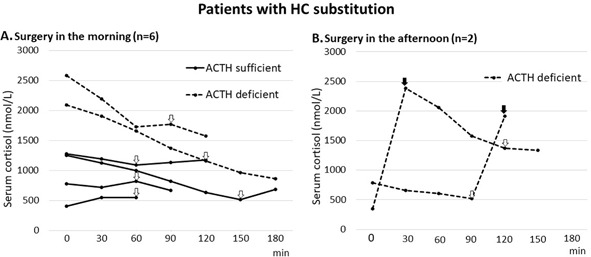
Serum cortisol levels in patients with perioperative hydrocortisone substitution, ACTH sufficient (*n* = 4) and ACTH deficient (*n* = 4), during pituitary surgery in the **a** morning (*n* = 6) and **b** afternoon (*n* = 2). Serum cortisol was measured from the start to the end of surgery. ACTH sufficient patients received 50 mg HC about 1 h before start of surgery. ACTH deficient patients received 100 mg at 6 AM, and those with surgery in the afternoon also received additional 50 mg during surgery, 8 h after the morning dose. Black arrows (⬇) indicate the first sample obtained after the 50 mg dose (**b**). White arrows (⇩) indicate the first sample obtained after intrasellar manipulation

All 4 patients had surgery in the morning, and at start of surgery the cortisol levels ranged from 407 to 1282 nmol/L (Fig. 3a). During the first part of surgery cortisol levels decreased, but after intrasellar manipulation the cortisol levels increased somewhat again, with (Δ) 7–170 nmol/L. In 1 of the 4 patients there was no prompt increase in the cortisol level, but an increase was noted in the second sample obtained after entrance into the sella (Fig. 3a).

#### ACTH deficient patients

The 2 patients who had surgery in the morning received 100 mg HC 2–3 h earlier and their cortisol levels started at a very high level and decreased gradually during the operation, from 2094 to 862, and 2582 to 1577 nmol/L, respectively (Fig. 3a). One of these patients with surgery in the morning showed some increase in cortisol levels with (Δ) 44 mmol/L after intrasellar manipulation.

The 2 patients who had surgery in the afternoon received HC 7–10 h earlier and started at cortisol levels of 347 and 787 nmol/L, respectively (Fig. 3). These 2 patients received additional 50 mg HC during the operation and the cortisol levels peaked at 2384 and 1914 nmol/L, respectively (Fig. 3b).

#### Premedication and anesthetics

Among the ACTH sufficient patients, 4 patients were given premedication with midazolam, a benzodiazepine, that reduces stress during surgery. Of the 4 patients, 3 did not receive HC substitution, and 2 of those had surgery in the afternoon (Table 1). Among the ACTH insufficient patients, 3 out of 4 were given midazolam.

Figure 1 and Table 1 show the doses of propofol (20 mg/mL) and remifentanil (50 µg/mL) used in each patient, 59–150 and 39–128 mL, respectively. We observed no correlation between these doses and the change in serum cortisol levels during surgery.

#### Postoperative cortisol function

Serum cortisol levels were measured each morning after surgery and 8 of 11 ACTH sufficient patients retained normal adrenal reserve with morning cortisol levels similar to before surgery. The remaining 3 patients, 2 with and 1 without perioperative HC substitution, had morning cortisol levels of 45, 53, and 121 nmol/L, respectively and were given oral HC replacement at discharge from the hospital. If the morning serum cortisol level was 200–400 nmol/L the patients received 10 + 10 mg HC, and with a morning cortisol level below 200 nmol/L the patients received 20 + 10 mg HC at discharge. We observed no correlation between the increase in cortisol during surgery and the postoperative HPA axis function. The patient without HC substitution had an increase in cortisol after intrasellar manipulation from 79 to 556 nmol/L, whereas the 2 patients who received 50 mg HC had increases from 407 to 553 nmol/L, and from 513 to 683 nmol/L, respectively. At follow-up about 3 months after surgery, 2 of the 3 patients had a normal ACTH stimulation test and HC replacement was ended. The third patient was followed elsewhere, and we do not have any follow-up data.

## Discussion

To the best of our knowledge this is the first study showing the serum cortisol levels obtained during surgery in ACTH sufficient patients without HC substitution and during routine HC substitution among ACTH deficient patients. A reason put forward for not giving ACTH sufficient patients HC replacement perioperatively is that it is easier to evaluate the postoperative ACTH function, as HC given in the afternoon may lead to falsely higher cortisol levels the morning thereafter. Further, as routinely early postoperative morning cortisol levels will immediately identify the need of HC substitution, the patient is not attributed to any risk [9]. At present, HC substitution in ACTH sufficient patients is not standard care in patients undergoing pituitary adenoma surgery. It has, however, been used in pituitary surgery due to the potential risk of surgical damage to the normal pituitary. For skilled neurosurgeons performing transsphenoidal surgery, that risk is however small in patients with noninvasive pituitary adenomas and in many centers HC substitution is only used in patients with preoperative subnormal ACTH function. In other centers, as ours, multiple doses of HC are still used in all patients [7, 10].

### ACTH sufficient patients without HC substitution

In the patients who did not receive any HC substitution and were operated in the afternoon, serum cortisol fell to levels of <50 nmol/L during the first part of the operation in nostrils and sinuses. Partly, this decline can be explained by the normal diurnal variation with the highest ACTH and cortisol levels early in the morning followed by a decline during the day. On the other hand, the very low values observed here, especially in the afternoon, may also be caused by premedication with midazolam, and the anesthetics propofol and remifentanil. Midazolam has been shown to reduce cortisol and ACTH during surgery [11]. It is well known that opioids as, e.g., sufentanil are 5–10 times as potent as fentanyl in large doses in abolishing the increase in plasma concentrations of cortisol during an open heart surgery [12]. Morphine has been shown to blunt the ACTH and cortisol response to CRH in vivo [13]. Similar as for benzodiazepines, the beneficial effect of remifentanil is to reduce the stress response during surgery [14]. However, the numbers are small in each subgroup and the study design does not allow for a clear distinction between anesthetic-induced low cortisol versus, e.g., a low normal cortisol concentration in the afternoon.

Whether propofol by itself reduces cortisol is more debated [15]. The amount of anesthetics used in the present study varied between the patients and no significant differences in doses appeared between the patients with and without HC substitution. In addition, there was no association between doses of anesthetics and the serum cortisol response among patients without HC substitution.

After intrasellar manipulation, serum cortisol levels increased in the next obtained sample(s) in 6 of 7 patients. In the only patient in whom no such increase was detected, surgery was shorter than in the others, and the patient was not followed as long after intrasellar manipulation. In the other two patients with operations in the afternoon, the increase in serum cortisol after intrasellar manipulation was somewhat lower, compared to the cortisol levels recorded in the patients operated in the morning. The reason is possibly the diurnal decline in ACTH/cortisol levels in the afternoon, with less responsiveness in the HPA axis, due to the hypothalamic suprachiasmatic biological clock.

### ACTH sufficient patients with HC substitution

In the 4 ACTH sufficient patients who received our standard 50 mg HC substitution before surgery, serum cortisol levels were stable at about 500–1300 nmol/L throughout surgery (Fig. 3a). Notably, these patients had a lower increase in cortisol levels after intrasellar manipulation compared to those ACTH sufficient patients without HC substitution (Fig. 3a, b). This may be due to the negative feedback on the HPA axis by the high serum cortisol levels achieved during HC substitution.

### ACTH deficient patients

In the 4 ACTH deficient patients, perioperative HC supplementation resulted in high circulating cortisol levels during surgery, up to about 2500 nmol/L (Fig. 3a, b). Despite these high cortisol levels, cortisol secretion after intrasellar manipulation may not have been totally depleted as one of the patients showed a small increase (Fig. 3a). However, that increase could also be due to the pulsatile secretion pattern of ACTH and cortisol [16]. Considering the high cortisol levels achieved several hours after 100 mg intravenous HC for patients with surgery in the afternoon, it may seem more appropriate to give them their usual oral HC morning dose and administer the stress dose just before the start of surgery.

Can the very low cortisol levels observed during surgery in ACTH sufficient patients without HC be harmful, and should we recommend HC substitution in all patients? The answer is probably no. The anesthetics used in the present study are also used in other types of surgery, and these patients, as ACTH sufficient, are not given HC substitution. The major perioperative risks in transsphenoidal surgery are cerebrospinal fluid leakage, hemorrhages, and thrombosis in the operative area. The risk of thrombosis has been suggested to be increased by ACTH and cortisol [17] but is not likely affected by a few hours of either low or supraphysiological cortisol levels, although this has not been explicitly studied. The patients in the present study had low cortisol levels for about 2 h during surgery, and after intrasellar manipulation cortisol levels were increased. In a previous report, no differences in blood pressure, pulse, nor in the dose of anesthetics needed during surgery, was shown between patients with and without perioperative HC substitution [18]. In the present study, the results of blood pressure and pulse were regulated by the dose of anesthetics and nothing abnormal was observed in any of the patients and there was no association between these parameters and the dose of anesthetics (Table 1).

Indeed, it is not clear whether the supraphysiological cortisol levels after 50 or 100 mg HC injections are beneficial or harmful. If the aim is to mimic the physiological cortisol levels, the results from this small study suggest that in transsphenoidal endoscopic pituitary surgery, the HC doses in ACTH insufficient patients could be reduced to 50 + 25 + 25 mg rather than being 100 + 50 + 50 mg of HC. This is less than suggested in a study comparing urinary cortisol levels during endoscopic pituitary adenoma operations in ACTH insufficient patients [19]. Interestingly, in ACTH sufficient patients, the pituitary surgery itself assures high perioperative cortisol levels during the second part of the operation.

## Conclusion

In endoscopic pituitary adenoma surgery among patients with normal ACTH function, cortisol levels increased after mechanical impact in the sella. To this mechanical impact the highest serum cortisol levels were observed in ACTH sufficient patients without HC replacement who had morning surgery. The same patient group had very low cortisol levels during the first part of surgery likely caused by one or both anesthetics propofol and remifentanil. Among ACTH insufficient patients supraphysiological cortisol levels were achieved with our routine intravenous HC substitution, which would advise us to reduce the HC supplementation.
